# Genomic and Functional Characterization of Lytic *Tlsvirus* Bacteriophages Targeting *Salmonella* Infantis Isolated from Poultry Farms in Ecuador

**DOI:** 10.3390/biology15030232

**Published:** 2026-01-26

**Authors:** Sandra Sevilla-Navarro, Ignacio Samuel Gómez-Cano, Ivette Castillo-Beckmann, Santiago Ballaz, Alexis Debut, Esteban Fernández-Moreira

**Affiliations:** 1 Centro de Calidad Avícola y Alimentación Animal de la Comunidad Valenciana (CECAV), Alquerias del Niño Perdido, 12539 Castellón, Spain; s.sevilla@cecav.org (S.S.-N.); i.gomez@cecav.es (I.S.G.-C.); 2 Facultad de Ciencias de la Salud “Dr. Enrique Ortega Moreira”, Universidad Espíritu Santo (UEES), Samborondón 0901952, Ecuador; iveacast@gmail.com; 3 Materials & Proceses Research Group, Universidad a Distancia de Madrid-UDIMA, 28010 Madrid, Spain; sballazg@gmail.com; 4 Departamento Ciencias de la Vida y de la Agricultura, Centro de Nanociencia y Nanotecnología, Universidad de las Fuerzas Armadas (ESPE), Sangolquí 171103, Ecuador; apdebut@espe.edu.ec; 5 Department of Cell Biology, School of Medicine, Universidad Complutense, 28040 Madrid, Spain

**Keywords:** *Salmonella* Infantis, bacteriophages, depolymerase, biofilm, biocontrol, genomics

## Abstract

*Salmonella* is responsible for millions of foodborne illnesses worldwide. The emergence of antibiotic-resistant *Salmonella* strains necessitates the development of alternatives for controlling this microorganism in the food supply chain. In Ecuador, *Salmonella* Infantis (*S*. Infantis) is the most frequently isolated serovar in poultry farms, poultry food products, and human infections. The objective of this study was to isolate and characterize lytic bacteriophages against a *S*. Infantis strain from poultry products in Ecuador to evaluate their potential for biocontrol. Three bacteriophages, GS71, GS156, and GS166, were isolated from chicken feces samples and showed short latent times (5–10 min), burst sizes of 205–231 PFU/cell, and stability up to 50 °C and pH = 10. Despite being isolated at different times and locations, they exhibited high genomic similarity (91.9–98.7%), reflecting the low diversity of Ecuadorian *S*. Infantis strains. VIRIDIC and phylogenetic analyses placed them within the *Tlsvirus* genus, showing conserved gene modules for replication, morphogenesis, and lysis. Putative endolysin and depolymerase genes were identified, supporting their anti-biofilm activity against biofilm-forming bacteria. Host range assays showed GS71 and GS166 lysed most *S*. Infantis field strains, whereas GS156 had a narrower spectrum linked to a unique polynucleotide kinase insertion. TEM confirmed Siphovirus-like morphology with icosahedral capsids (~55 nm) and long non-contractile tails. No genes associated with lysogeny, virulence, or antibiotic resistance were found. These findings support GS71, GS156, and GS166 as safe and effective candidates for bacteriophage cocktails targeting multidrug-resistant *S*. Infantis in poultry production.

## Simple Summary

*Salmonella* causes millions of foodborne illnesses each year, and the rise of strains that no longer respond to antibiotics makes it necessary to find new ways to control this bacterium in the food chain. In Ecuador, the type of *Salmonella* most commonly found in poultry farms, poultry meat, and human infections is *Salmonella* Infantis. In this study, we searched for viruses that specifically infect and destroy this bacterium, known as bacteriophages, to evaluate whether they could help improve food safety. We isolated three bacteriophages from chicken farms in different regions of Ecuador and found that they acted quickly, produced many new viral particles, and tolerated high temperatures and alkaline conditions. Although the bacteriophages came from different places and times, they were genetically very similar, reflecting the low diversity of *Salmonella* Infantis in Ecuador. Two of them were able to kill most of the tested bacterial strains. None carried genes associated with harmful effects. These results show that the three bacteriophages are safe and promising candidates for use in mixtures designed to reduce antibiotic-resistant *Salmonella* Infantis in poultry production.

## 1. Introduction

Diseases linked to *Salmonella* infection are of major worldwide concern. Globally, non-typhoidal *Salmonella* is thought to be responsible for 155,000 deaths and 99.8 million illnesses each year [1]. The majority of human infections stem from eating undercooked or contaminated food, with poultry being the main source [2]. In recent decades, there has been a notable increase in the prevalence of multidrug-resistant (MDR) *Salmonella* worldwide, with *Salmonella* enterica serovar Infantis (*S*. Infantis) emerging as one of the most concerning serovars [3]. In Europe, *S*. Infantis is among the top five European Union-acquired serovars involved in human Infections [4]. Moreover, it is by far the dominant serovar in the “broilers–broiler meat” category and ranks among the top four serovars across all considered food-animal sources [4]. A similar trend is observed in Ecuador, where *S*. Infantis is most prevalent in poultry farms, food, and humans, which show several patterns of antibiotic resistance [5].

Given the ongoing rise in antimicrobial resistance, bacteriophages, viruses of bacteria, have gained attention as potential alternatives and as tools for food biocontrol [6]. Bacteriophages infect bacteria with high specificity and lyse them upon completing their replication cycle. Given that 99% of bacteria survive in biofilms in the natural world [7], it is advantageous for bacteriophages to have depolymerases that can target the biofilms that serve as bacterial refuges and defenses. Interest in bacteriophages as an alternative to antibiotics on farms [8] has skyrocketed since the FDA approved the first bacteriophage product for food processing plants in 2006 [9]. The criteria for selecting bacteriophages for application in agricultural facilities are that they display a strictly lytic infection cycle, with no genes associated with lysogeny, carry antibiotic resistance genes or have virulence genes. Although bacteriophage therapy presents an alternative to antibiotics for controlling *Salmonella* proliferation in dynamic farm environments [10], a limited number of studies have been published regarding the use of bacteriophages in controlling specifically *S*. Infantis [11,12,13,14]. In this vein, this study was focused solely on candidate bacteriophages specific to *S*. Infantis due to its increasing prevalence as an emerging human MDR pathogen [3]. This study provides one of the first characterizations of Tlsvirus bacteriophages active against Ecuadorian *S*. Infantis isolates and offers a comprehensive approach that integrates anti-biofilm phenotyping with comparative genomic analysis for *S*. Infantis phages. This integrated perspective provides new insight into their potential as safe and effective biocontrol agents in the food production chain.

The purpose of this study was to isolate and comprehensively characterize lytic bacteriophages targeting an *S*. Infantis strain isolated in Ecuador, evaluating their biological properties, genomic safety, anti-biofilm potential, and phylogenetic relationships, for potential use as biocontrol agents in the food supply chain.

## 2. Materials and Methods

### 2.1. Bacterial Strains and Culture Conditions

*S*. Infantis U1068 was isolated from chicken carcasses obtained in Quito (Ecuador) and used as a host for bacteriophage isolation. Additionally, strains of *S*. Infantis ATCC 51741, *Salmonella* enterica serovar Enteritidis (*S*. Enteritidis) ATCC 13076, and *Escherichia coli* DH5α were used to visualize the morphology of the plaques of bacteriophages. Bacteria were incubated until they reached the exponential phase (OD_600_ = 0.5–0.6). One hundred microliters (µL) of the *Salmonella* culture in the exponential growth phase were mixed with 100 µL of lysate containing 10^3^ PFU/mL. The mixture was incubated at 37 °C for 5 min and subsequently cultured using the double agar layer technique. The plaques were incubated overnight at 37 °C. Each strain was cultivated at 37 °C and 150 rpm in liquid Trypto-Casein Soy Broth (TSB) medium (Difco™, BD, Franklin Lakes, NJ, USA). Solid medium cultures were carried out on TSB plates containing 1% agar (Difco™, BD, USA). The *S*. Infantis strain used to propagate the bacteriophages was grown at 37 °C and 150 rpm in liquid TSB medium with 0.2% MgSO_4_ (MilliporeSigma, St. Louis, MO, USA). 

### 2.2. Isolation and Propagation of Bacteriophages

*Salmonella* bacteriophages were isolated using a modified version of a previously published method [15]. A total of forty-eight chicken feces and wastewater samples were collected from rural areas of the Ecuadorian coast between 2021 and 2023. The samples were enriched by diluting 3 g or 3 mL of the sample in a 1/10 ratio with TSB culture medium that included 0.2% MgSO_4_. Next, 1 mL of exponential-phase *S*. Infantis U1068 (OD_600_ = 0.5–0.6) and ceftriaxone (CRO) were added at a final concentration of 10 ppm. The enriched samples were incubated overnight at 37 °C and 150 rpm. The samples were centrifuged at 4000 rpm for 30 min at 4 °C to separate bacteria and solid residues. The supernatant was filtered through a 0.22 µm syringe filter (MilliporeSigma, Burlington, MA, USA), and chloroform was added at a ratio of 1/100. Ten microliters of sample was mixed with 100 µL of *S*. Infantis, and the solution was cultured by the double-layer agar (DLA) method, using TSB with 0.2% MgSO_4_ and 0.5% agar as the upper semisolid medium. The plates were incubated at 37 °C and then examined for the presence of lysis plaques (circular clearing spaces) in the bacterial lawn. An isolated plaque from each sample was pricked with a pipette tip and suspended in 300 μL of TSB with 0.2% MgSO_4_. This process was repeated three times to ensure uniformity of the isolated bacteriophage. Purified bacteriophages were propagated by inoculating a culture of *S*. Infantis U1068 at an OD_600_ of 0.2, and the absorbance was measured every 30 min until a steady drop in optical density was attained. The lysates were centrifuged to eliminate bacteria, and chloroform was added at a 1% final concentration to the supernatant. The lysates were stored at 4 °C. A sample of each lysate was titrated by the duplex technique, and the concentration in PFU/mL was recorded.

### 2.3. DNA Extraction and Genomic Sequencing

Bacteriophage DNA was extracted using the phenol-chloroform method, as previously described [16]. A 20 mL volume of high-concentration bacteriophage lysate (10^8^–10^9^ PFU/mL) was centrifuged at 50,000× *g* for one hour at 4 °C in an ultracentrifuge (Thermo Fisher Scientific, Waltham, MA, USA) using an AH-650 rotor (Thermo Fisher Scientific, Waltham, MA, USA). The pellet was suspended in 450 μL of Buffer SM (50 mm Tris-HCl, pH 7.5; 100 mm NaCl; 8 mm MgSO_4_). The suspension was treated with DNase and RNase to final concentrations of 0.01 U/μL and 0.05 mg/mL, respectively. The mixture was incubated at 37 °C for one hour. The mixture was then treated with EDTA, SDS, and proteinase K, and incubated at 60 °C for one hour. After adding an equivalent volume of phenol-chloroform-isoamyl (25:24:1), the mixture was centrifuged for 5 min at 6000 rpm. The aqueous phase was collected, an equivalent volume of chloroform was added, and the centrifugation process was repeated. After gathering the upper aqueous phase in a tube, potassium acetate and 100% ethanol were added in a 2.5 ratio. The mixture was incubated at −20 °C overnight. The mixture was centrifuged at 13,000× *g* for 20 min at 4 °C. The DNA pellet was washed twice with 70% ethanol. Finally, the DNA was allowed to dry at room temperature, resuspended in ultrapure water, and stored at −20 °C.

### 2.4. CsCl Gradient Purification

Bacteriophage purification by CsCl gradient was conducted as previously described [17], with minor modifications. An ultracentrifuge (Sorvall WX 80) with a TH-641 rotor (Thermo Fisher Scientific, Waltham, MA, USA) was used to centrifugate 400 mL volume of high-concentration bacteriophage lysate (10^8^–10^9^ PFU/mL) at 50,000× *g* for one hour at 4 °C. To remove any remaining debris, the pellet was resuspended in 2 mL of Buffer SM (50 mm Tris-HCl, pH 7.5; 100 mm NaCl; 8 mm MgSO_4_) and centrifuged at 10,000× *g* for 5 min in a microcentrifuge. The resulting suspension was placed on a CsCl gradient composed of layers with densities of 1.3, 1.5, and 1.7 g/mL. The gradients were centrifuged in an AH-650 rotor at 100,000× *g* for 2 h at 4 °C. The bluish band between the 1.5 and 1.7 g/mL layers was collected and titrated using the double-layer method.

### 2.5. Host Range Determination

To evaluate the host range of the bacteriophages, a total of 29 *Salmonella* isolates from Spanish poultry farms previously characterized and obtained from the repository of Centro de Calidad Avícola y Alimentación Animal de la Comunidad Valenciana (Spain) were analyzed [13]. This study focused particularly on *S*. Infantis strains, which showed 100% resistance to at least one antibiotic and more than 70% multidrug resistance (MDR), and included isolates from different years: 2023 (*n* = 10; Minf 1–10), 2018 (*n* = 10; Minf 11–20), and 2013 (*n* = 9; Minf 21–29) [13].

The ability of the bacteriophages to infect these 29 strains was assessed using the spot test assay. Bacterial strains were prepared via the DLA technique. Briefly, 200 µL of bacterial inoculum in Luria–Bertani (LB acc. Miller) medium (Sharlau, Barcelona, Spain) at an OD_600_ of 0.2 (~10^8^ CFU/mL) were mixed with 5 mL of semi-solid LB medium (supplemented with 0.6% agar-agar, VWR Chemicals, Barcelona, Spain) and poured onto solid LB agar plates (1.5% agar). The plates were dried under a laminar flow hood for ten minutes. Subsequently, 10 µL of each bacteriophage was spotted onto the double-layer agar surface. The plates were incubated at 37.5 °C for 24 h, after which bacteriophage-induced lysis zones were evaluated on the bacterial lawns [13,18].

### 2.6. Efficiency of Plating (EOP)

Bacteriophage lysates that showed a positive spot were plated on each bacterial strain using the DLA method. The plaques obtained were counted, and the efficiency of plating (EOP) (average PFU in the isolation bacteria/average PFU in the host bacteria) of each bacteriophage on each host strain was calculated. The EOP of each bacteriophage on each host bacteria, relative to the isolation host, was classified according to the following criteria: high production EOP ≥ 0.5; medium production 0.1 ≤ EOP < 0.5; low production 0.001 < EOP < 0.1 and no production EOP ≤ 0.001 [19].

### 2.7. Stability at pH and Temperature Ranges

The thermal stability range of the bacteriophages was assessed by incubating 1 mL of a 10^8^ PFU/mL suspension at different temperatures (4 °C, 25 °C, 37 °C, 50 °C, 60 °C, 70 °C, and 80 °C) for one hour. The suspensions were rapidly titrated after incubation using the DLA method. To assess the pH stability range of the bacteriophages, 0.1 mL of 10^8^ PFU/mL lysate of each bacteriophage was diluted in 0.9 mL of SM buffer, which was made using laboratory-grade chemicals at different pH values (from 3 to 12), and incubated at 37 °C for 1 h. The suspensions were rapidly titrated after incubation using the DLA method. The experiments were run in independent triplicates.

### 2.8. One-Step Growth Curve

Bacteriophage burst size and lag period were determined using the one-step growth curve assay with modifications [17]. Bacteriophage lysate (0.1 mL) was added to 0.9 mL of *S*. Infantis U1068 during the mid-exponential growth phase (OD_600_ = 0.5–0.6, approximately 5 × 10^8^ bac/mL) to reach a concentration of 5 × 10^7^ PFU/mL, resulting in an MOI of 0.01. The mixture was incubated for 5 min at 37 °C and then centrifuged at 13,000× *g* for 2 min. The pellet was resuspended in 1 mL of TSB (0.2% MgSO_4_) and then diluted to 10–4 in 30 mL of TSB (0.2% MgSO_4_). The mixture was incubated at 37 °C. Samples of 100 µL were taken at 0, 5, and 10 min, and then every ten minutes for 60 min. The samples were titrated by the DLA method and the concentration of PFU/mL recorded at each point. The time interval between infection to the first significant increase in PFU/mL in the culture was designated the latent period. The burst size was calculated as the average PFU/mL of the last three maximum points of the experiment divided by the average PFU/mL of the latent time. The experiments were run in triplicate, and the data were plotted on a curve of PFU/mL vs. time in minutes.

### 2.9. Lytic Activity in Planktonic Culture

Bacteriophage lytic activity assays in planktonic cultures were conducted as previously described [20], with modifications. Stationary phase *S*. Infantis U1068 was diluted to OD_600_ = 0.1 (approximately 1 × 10^8^ CFU/mL) in TSB (0.2% MgSO_4)_ and mixed with bacteriophage lysates, obtaining different MOIs (10, 1, 0.1, and 0.01) and a final volume of 10 mL. A *S*. Infantis culture at OD_600_ = 0.1 and TSB medium (0.2% MgSO_4_) were used as positive and negative controls, respectively. The OD of the cultures was measured in a spectrophotometer and recorded every hour for 8 h. Experiments were performed in triplicate, and data were plotted as an OD_600_ vs. time curve in hours.

### 2.10. Transmission Electron Microscopy (TEM) of Bacteriophages

A transmission electron microscope (TEM) Tecnai G2 Spirit Twin TEM (FEI Company, Hillsboro, OR, USA) operated at 80 kV and equipped with an Eagle 4k HR camera was used to identify the bacteriophages. Samples were dropped on Formvar-carbon 300× mesh grids, excess water was removed by using a paper filter, then stained using phosphotungstic acid (PTA) at 2% for 10 s. The purified bacteriophages were classified according to Ackermann’s criteria [21].

### 2.11. In Silico Genomic Characterization of the Bacteriophages

Raw sequencing data were processed using FastQC v0.11.9 and fastp v0.20.1 for quality control and adapter removal [22,23,24]. The filtered reads were then assembled with SPAdes v4.0.0 (only-assemble mode) using the following k-mers list: 33, 55, 77, 99 and 127 [25,26]. The assembled contigs were analyzed using Geneious Prime v2025.0.3, and for each bacteriophage, the contig with the highest coverage and length was selected as the putative genome. BLAST (online version) was performed against the nucleotide collection database to find the closely matching bacteriophages to assign a preliminary taxonomic classification [13]. Genome rearrangement was performed manually using Progressive Mauve, guided by the reference sequence (RefSeq) of the type-bacteriophage from the *Tlsvirus* genus [13,27,28]. To ensure accuracy in defining the genomic termini, PhageTerm was additionally used to validate the start and end positions of each genome [29].

Read mapping was performed using BBMap.sh v38.84 to evaluate coverage of the trimmed reads against the assembled contigs [30]. The assembly was further refined using Pilon v1.20 [30]. With the corrected genomes, a multiple sequence alignment was conducted using ClustalW implemented in Geneious Prime v2025.0.3 [31]. For structural and functional annotation, Pharokka v1.4.1 was used with default parameters, and each sequenced coding region was associated with a Phrog functional group [32]. Protein sequences were manually cross-checked with BLASTp searches [33,34].

Pharokka and PhageLeads were used to assist in the prediction of therapeutic suitability, while Abricate was used to identify antimicrobial resistance and virulence genes [35,36]. Phage lifestyle prediction was performed using PhaTYP from PhaBOX v.2.0 [37], and the presence of depolymerase enzymes was predicted using the DePolymerase Predictor (DePP, web version 1.0.0) machine learning tool, considering proteins with a probability greater than 90% as potential depolymerases [38].

The taxonomic classification of the bacteriophages was assessed using VIRIDIC v1.1, which calculates pairwise intergenomic similarities and presents the results as a heatmap and similarity matrix [39]. Thresholds of 95% for species-level and 70% for genus-level classification were applied. The bacteriophage Warwickvirus SLUR29 (accession number NC_054895.1), from a different genus within the same family (subfamily Tempevirinae), was included as an outgroup in both VIRIDIC and phylogenetic analyses.

To support these results, a phylogenetic tree was constructed based on the large terminase subunit gene (*terL*). Nucleotide sequences were aligned using Clustal Omega v1.2.2 [32] and the tree was inferred using IQ-TREE v1.6.12 under the maximum likelihood method with 1000 bootstrap replicates [40]. The tree was visualized using iTOL v6 [41] and included the same outgroup to root the topology. In addition, a structural comparison was made using Clinker [42], including representative genomes from the same genus infecting *Escherichia coli* and *Citrobacter*.

## 3. Results

### 3.1. Bacteriophage Isolation

**Figure 1:**
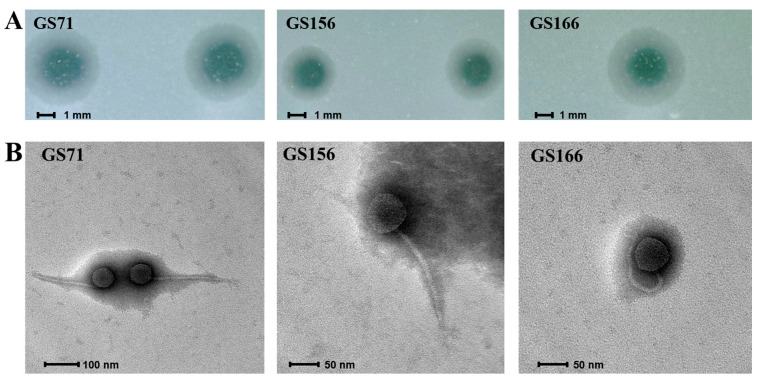
Morphological characteristics of the bacteriophages. (**A**) Morphology of the plaques of bacteriophages GS71, GS156, and GS166 produced on a lawn of *S*. Infantis. (**B**) Micrographs of the bacteriophages stained with 2% PTA.

A total of 24 bacteriophages capable of infecting *S*. Infantis were isolated from chicken feces collected at poultry farms located at various sites across the Guayas estuary—Guayaquil, Pedro Carbo, Milagro, Daule, Naranjal and El Empalme—in the province of Guayas (Ecuador). These locations are situated at least 40 km apart from one another. Three representatives: GS71 (accession number: PV090992.1), GS156 (accession number: PV153712.1, and GS166 (accession number: PV090993.1) were selected for in-depth genetic and phenotypic characterization because they had lytic halos that suggested depolymerase activity. The lytic halo diameters (mean ± SD, *n* = 10 replicates) using *S*. Infantis U1068s as the host were 2.9 ± 0.22 mm for GS71, 2.2 ± 0.27 mm for GS156, and 2.6 ± 0.22 mm for GS166 (Figure 1). To reduce the possibility of clonality, we also tried to make sure the phage isolates were as far apart in both space and time as possible. Phage GS71 was thus isolated in Naranjal in December 2021, GS156 in Pedro Carbo in February 2021, and GS166 in El Empalme in December 2022. The three locations are at least 150 km apart.

### 3.2. Bacteriophage Characterization

Three lytic bacteriophages, GS71, GS156, and GS166, were isolated from poultry-derived samples and propagated using *S*. Infantis U1068s as the host strain. All isolates produced clear plaques with surrounding halos, indicative of potential depolymerase activity. Plaque size measurements revealed highly similar lytic phenotypes among the three phages, with mean diameters of 2.81 ± 0.17 mm for GS71, 2.74 ± 0.20 mm for GS156, and 2.81 ± 0.11 mm for GS166. These results suggest that, under the tested circumstances, the three phages exhibit similar lytic behavior and infection dynamics (Figure 1A).

The purified bacteriophages were analyzed by TEM and classified according to Ackermann’s criteria [21]. Micrographs revealed that all three bacteriophages possessed an icosahedral capsid and a long, non-contractile tail. They were classified within the class *Caudoviricetes* and displayed a Siphovirus-like morphology (Figure 1B).

### 3.3. Host Range and Efficiency of Plating Determination

The lytic range of the three bacteriophages was determined in two commercial strains of *S*. enterica. All three bacteriophages had lytic activity on *S*. Infantis U1068s and commercial *S*. Infantis ATCC 51741, *S*. Enteritidis ATCC 13076, and no lytic activity on *Escherichia coli* DH5α. However, the EOP analysis revealed that none of the bacteriophages were able to form plaques on these commercial strains, except for the isolation host.

**Figure 2:**
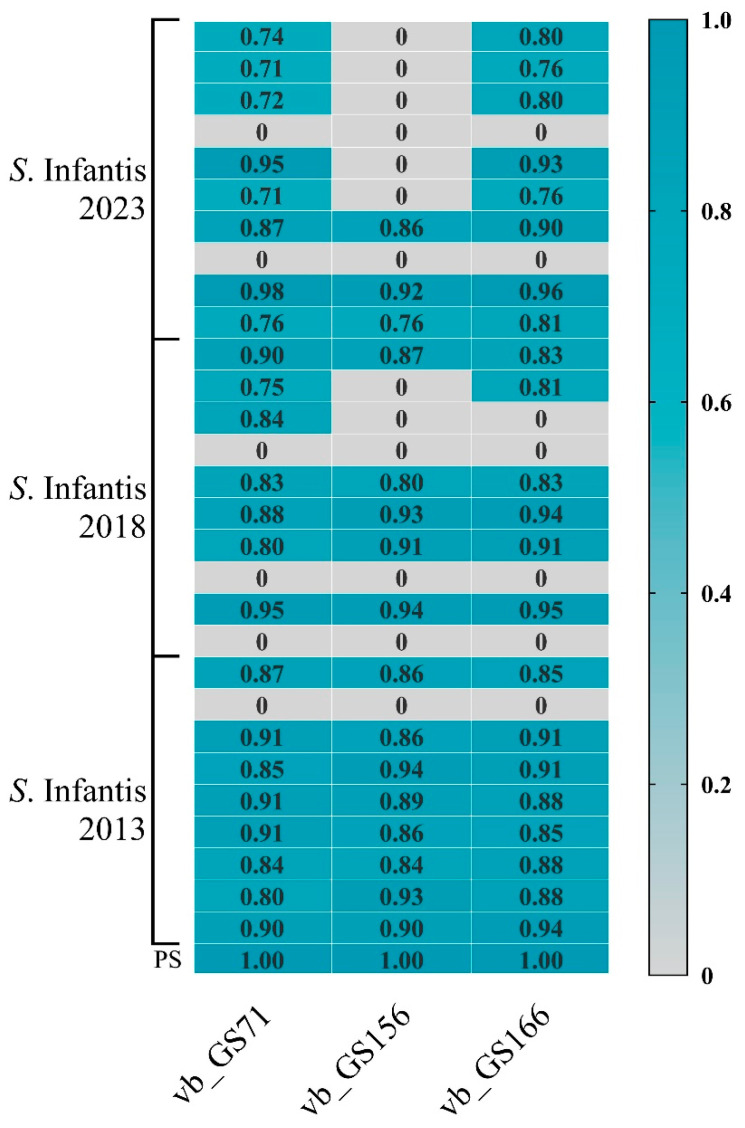
Host range and efficiency of plating (EOP) of bacteriophages GS71, GS156, and GS166 against 29 *S*. Infantis field strains and their propagation strain (PS). EOP was calculated relative to the bacteriophage titer obtained on its own PS. Strains are grouped by year of isolation (2023, 2018, and 2013), and grey cells indicate absence of productive infection (EOP = 0). With a maximum of 1, blue intensity indicates increasing EOP values. The reference host for normalization was the propagation strain (PS).

A total of 87 assays with bacteriophages GS71, GS156, and GS166 were conducted in *S*. Infantis isolated from Spanish poultry farms. Productive infection was defined by the presence of a measurable EOP value, indicating both bacterial susceptibility and effective bacteriophage replication. Sixty-one (70.1%) of the 87 bacteriophage-strain combinations that were investigated had detectable EOP values. Accordingly, 23 of the 29 strains (79.3%) were susceptible to at least one of the bacteriophages. Bacteriophage GS71 infected 23 strains, with EOP values ranging from 0.71 to 1.00. GS166 infected 22 strains, with EOP values between 0.76 and 1.00. GS156 showed the narrowest host range, infecting 16 strains, with EOP values ranging from 0.76 to 0.94. In all cases, most productive infections yielded EOPs greater than 0.85, indicating high replication efficiency in the susceptible *S*. Infantis isolates (Figure 2).

### 3.4. One-Step Growth Curves

**Figure 3:**
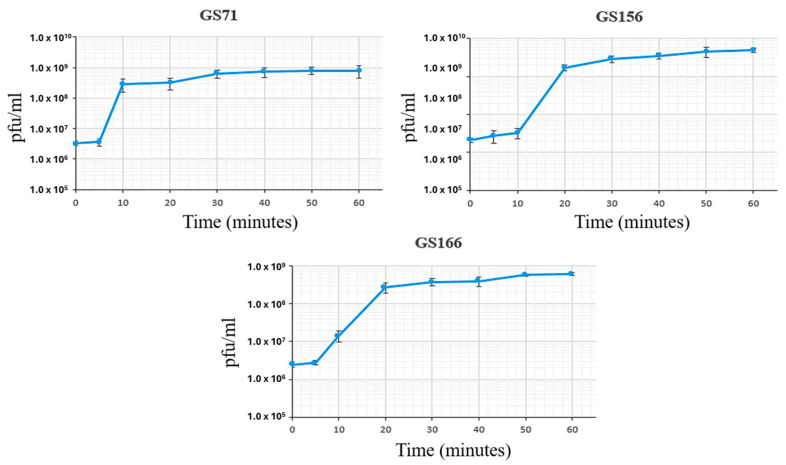
One-step growth curves of bacteriophages GS71, GS156, and GS166. Data presented are the average of three independent experiments, along with the corresponding standard deviation. X-axis: time in minutes; Y-axis: PFU/mL.

A one-step growth curve assay was carried out to determine the lag time and burst size of the isolated bacteriophages. All three bacteriophages had short lag times: 5 min for GS71 and GS166, and 10 min for GS156. The burst sizes of GS71, GS156, and GS166 were 231, 205, and 215 PFU/cell, respectively (Figure 3).

### 3.5. Thermal and pH Stability Profiles of the Phages

**Figure 4:**
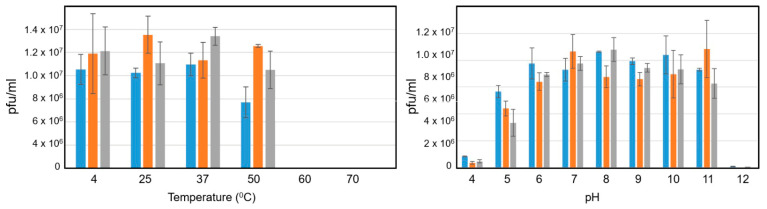
Thermal stability and pH tolerance of bacteriophages GS71 (blue), GS156 (orange), and GS166 (gray). Plaque-forming units per milliliter (pfu/mL) were used to measure phage viability after exposure to various pH levels (**right panel**) and temperatures (**left panel**). Three separate replicates of each condition were examined. Standard deviation is shown by error bars.

Bacteriophages GS71, GS156, and GS166 were tested for stability at different pH and temperature levels. At temperatures between 25 °C and 37 °C, all three phages remained highly viable; at 60 °C, there was a noticeable decrease, and at 70 °C, there was total inactivation. Similarly, extreme acidic (pH 4) and alkaline (pH 12) conditions greatly decreased infectivity, while neutral to slightly alkaline pH values (pH 7–8) were optimal for phage viability. The tolerance profiles of GS71, GS156, and GS166 were similar, though GS156 demonstrated somewhat greater resilience at pH 6 and 50 °C (Figure 4).

### 3.6. Genomic Analysis of the Bacteriophages

The complete genomes of bacteriophages GS71, GS156, and GS166 measured 48,587 bp, 48,552 bp, and 48,069 bp, respectively, with a GC content ranging from 41.7% to 41.8%. Annotation predicted 79 coding sequences (CDS) in both GS71 and GS156, and 76 in GS166. There were no tRNA genes or pseudogenes found (Table 1).

**Figure 5:**
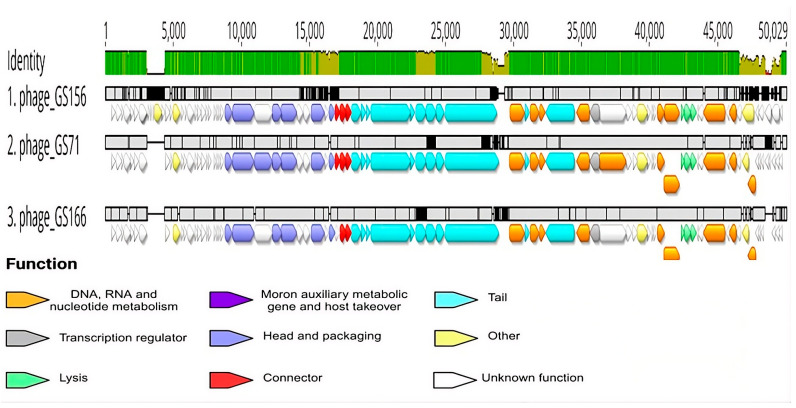
Whole-genome alignment of bacteriophages: (1) GS156, (2) GS71, and (3) GS166. Generated using Geneious Prime 2024.0.7. The upper panel shows pairwise sequence identity across the aligned genomes, with drops in the curve indicating regions of variability. Predicted coding sequences, identified by Pharokka, are represented as colored arrows based on functional groups as defined by PHROG. Alignment was performed using MAFFT v1.4.0.

Gene content across the three genomes followed a conserved modular structure, with genes grouped into distinct regions involved in replication, structural assembly, lysis, and packaging. Pharokka identified tail-related proteins in all three bacteriophages, and DePP detected one candidate depolymerase per genome, each with a probability greater than 90% (Figure 5).

The gene was located on the positive strand in all cases, with slight positional variation: ORF44 in GS71 (23,637–27,497 bp), ORF49 in GS156 (24,951–28,727 bp), and ORF43 in GS166 (23,621–27,481 bp). PhageTerm analysis confirmed that all three bacteriophages utilize a Headful (pac) type 1 genome packaging mechanism (Table 2).

Additionally, PhageLeads and PhaTYP analyses predicted that all phages followed a strictly lytic infection cycle, with no evidence of temperate markers or virulence factors. Furthermore, ABRicate screening confirmed the absence of antimicrobial resistance genes across all three genomes, reinforcing their suitability for therapeutic or biocontrol applications.

**Figure 6:**
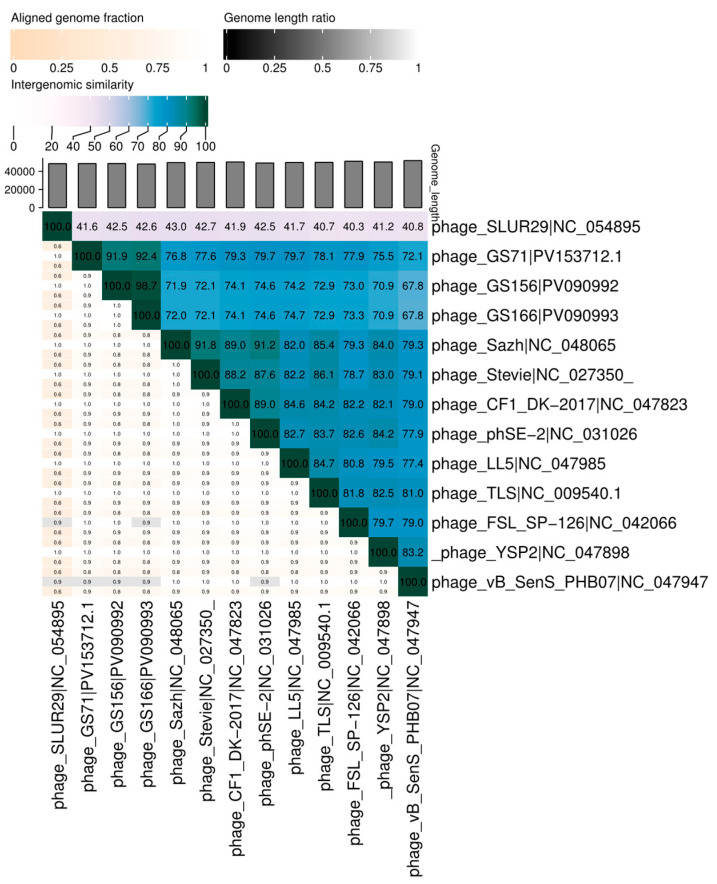
Intergenomic similarity heatmap generated by VIRIDIC. The matrix shows pairwise genomic similarity between the studied bacteriophages and reference genomes. Values above 70% indicate genus-level relatedness.

VIRIDIC analysis showed that bacteriophages GS71 and GS166 shared 98.7% intergenomic similarity. GS156 exhibited slightly lower similarity values with GS71 (92.4%) and GS166 (91.6%). All three bacteriophages showed values above the 70% genus-level threshold when compared to reference members of the *Tlsvirus* genus. In contrast, the outgroup bacteriophage SLUR29 displayed values below 75%, confirming its genomic divergence from the studied bacteriophages (Figure 6).

**Figure 7:**
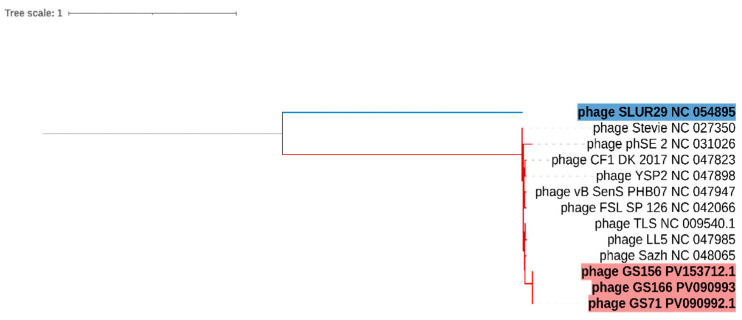
Phylogenetic tree based on *terL* gene sequences of bacteriophages GS71, GS156, and GS166, along with reference bacteriophages. The tree was constructed using the maximum likelihood method, rooted at the midpoint, and visualized with iTOL v.6. Bacteriophages identified in this study are highlighted in red, the outgroup bacteriophage SLUR29 is shown in blue, and reference bacteriophages are shown using the default branch color.

The phylogenetic tree clustered GS71, GS156, and GS166 into a single monophyletic clade within the *Tlsvirus* genus (Figure 7). These bacteriophages are grouped closely with the type of bacteriophage TLS, whereas SLUR29 was placed in a distant branch, consistent with its classification in a different genus of the subfamily *Tempevirinae*.

**Figure 8:**
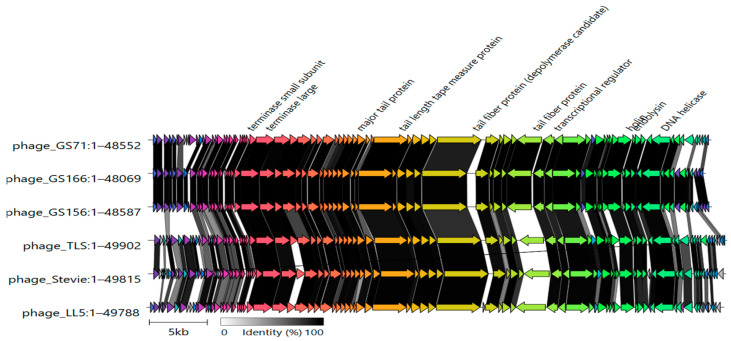
Comparative genomic organization of bacteriophages GS71, GS156, GS166, and related reference phages generated using Clinker. Genes are represented as arrows indicating transcriptional direction. Genes sharing sequence similarity are shown in the same color, and gray shading between genomes indicates regions of sequence homology. Conserved functional modules, including terminase, tail morphogenesis, and DNA replication genes, are indicated.

Comparative genome alignment using Clinker revealed conserved synteny across the three bacteriophage genomes. Structural, replication, lysis, and packaging modules were preserved across all isolates. Annotated conserved genes included terminase, tail fiber, DNA polymerase, and lysozyme, among others. Sequence variability was mainly located in short ORFs or regions encoding hypothetical proteins (Figure 8).

## 4. Discussion

The main finding of the study was that the three lytic bacteriophages (GS71, GS156, and GS166) collected between 2021 and 2023 from diverse sources and locations in Ecuador exhibited high genomic similarity, with intergenomic identities ranging from 91.9% to 98.7%. The predominance of a single clonal lineage (ST32) carrying a pESI-like megaplasmid [5] illustrates the remarkably homogeneous genomic profile of *S*. Infantis in Ecuador. The strong epidemiological connectivity between these environments is demonstrated by the consistent detection of this clone in poultry farms, throughout the poultry production and distribution chain, and in clinical isolates from human infections. The *Ecuadorian S.* Infantis population’s clonal structure suggests a low level of bacterial genomic diversity, which limits the evolutionary space accessible to related bacteriophages. Because of this, phages that circulate in this homogeneous host background have a tendency to converge toward similar genomic solutions, which eventually reduces inter-phage divergence. This aligns with studies showing conserved bacteriophage populations in ecologically stable host communities of bacteria [43]. Low genomic divergence among *S*. Infantis strains has significant clinical relevance, primarily because it indicates the rapid dissemination of highly successful pathogenic clones, particularly those carrying a multidrug resistance megaplasmid (pESI-like) [3] in poultry and food production, as well as in human infections in Ecuador [43]. The emergence of antibiotic-resistant *Salmonella* strains in Ecuador [5] has renewed interest in bacteriophages as targeted biocontrol [13,14] agents, particularly those found in this investigation.

Due to the presence of different *S*. Infantis clades circulating globally, it is essential to have previously isolated and well-characterized bacteriophages that are effective against region-specific bacterium clades. According to current ICTV species demarcation criteria (95% identity), GS71 and GS156 qualify as distinct new species, while GS166 (98.7% like GS71) falls within the same species as GS71. VIRIDIC and phylogenetic analysis of the *terL* gene placed all three bacteriophages within a monophyletic cluster of the *Tlsvirus* genus, supported by high sequence similarity to known members and clear distinction from the outgroup bacteriophage SLUR29. Whole-genome alignments using MAFFT revealed strong nucleotide conservation in central regions, while the 5′ and 3′ termini were more variable, containing short ORFs or hypothetical proteins likely reflecting modular rearrangements typical of tailed bacteriophages. Comparative synteny analysis with Clinker showed conserved gene modules associated with DNA replication, morphogenesis, lysis, and transcriptional regulation, reflecting conserved genomic architectures among those of bacteriophages infecting *Citrobacter* and *E. coli* [44,45,46].

All three bacteriophages encode a putative depolymerase gene within their tail modules. While depolymerase prediction was initially based on high-confidence DePP scores (>90%), this annotation is further supported by the localization of these proteins within the tail morphogenesis region and their annotation as tail spike or tail fiber proteins, a domain architecture commonly associated with phage-encoded depolymerases. These enzymes likely accounted for the pronounced halos observed around lysis plaques, a hallmark of capsular degradation. Such enzymes are associated with enhanced activity against biofilms and increased penetration of mucosal surfaces [47,48,49]. This phenotype was consistent across all three bacteriophages in vitro.

Notably, GS156 exhibited a narrower host range and lower EOP compared to GS71 and GS166. While GS71 and GS166 lysed 23 and 22 *S*. Infantis strains, respectively (EOP > 0.85), GS156 lysed only 16 strains with reduced efficiency. These included poultry isolates from both Ecuador and Spain, suggesting GS156 retains infectivity across geographic lineages. Its reduced host range may stem from subtle genetic differences or the presence of a unique genomic insertion encoding a polynucleotide kinase (PNK), absent in GS71 and GS166. This enzyme is involved in nucleic acid metabolism and may help bacteriophages evade host defenses [50] but could also impose metabolic costs that reduce infectivity.

Morphologically, TEM revealed icosahedral capsids (approximately 55 nm) and long, non-contractile tails, consistent with the Siphovirus morphotype in Caudoviricetes. While bacteriophages with smaller capsids (e.g., Podoviruses) may diffuse more efficiently in biofilms or mucus, Siphoviruses can still exhibit anti-biofilm activity, especially when carrying depolymerases [49,51]. The strong plaque halos support their potential to degrade extracellular matrices.

Of the three sequences, two represent new species (GS71 and GS156), while GS166 has a genomic similarity greater than 98.0% with respect to GS71. Therefore, we can only assign two new species, and the bacteriophage with the highest similarity to the previous one will retain its original “organism_name” but will be classified within the first described species. Although bacteriophages GS71 and GS166 share 98.7% genetic similarity, they exhibit a 7% variation in their one-step growth curves. This difference may be explained by minimal variations in promoters or regulatory sequences, which could affect replication timing, assembly efficiency, or lysis dynamics. Additionally, mutations in endolysin, holin, and other enzymes involved in bacteriophage release may alter burst size or lysis time. Differences in host interaction or genomic rearrangements were ruled out, as both bacteriophages display similar host range profiles, and no such genomic differences were observed.

Genomic safety and therapeutic suitability were assessed using a complementary in silico framework combining ABRicate for antimicrobial resistance and virulence screening, PhageLeads for therapeutic assessment, and PhaTYP for lifestyle prediction. No temperate markers, virulence genes, or antibiotic resistance determinants were found in the genomes of GS71, GS156, or GS166, confirming their strictly lytic nature and genomic safety—key prerequisites for biocontrol applications [52,53,54]. The genomes also lacked tRNA genes, a common feature in small lytic bacteriophages that rely on the host’s translational machinery and are considered suitable for synthetic biology or production platforms [55]. Their small size makes them suitable for decreasing their encapsidation capacity and increasing their assembly efficiency in cell-free production systems. This characteristic may be useful in future biotechnological applications [56].

The lack of tRNA is associated with bacteriophages with a host-dependent on transcription machinery [57]. Although these in silico analyses are reassuring and widely used for preliminary phage selection, they do not fully substitute for in vivo safety and efficacy evaluations, which will be required prior to practical application.

The *Tlsvirus* genus is currently the best characterized bacteriophage genus known to utilize TolC, an antibiotic and toxin secretory channel protein, as a coreceptor, along with lipopolysaccharide. *Tlsvirus* phages attach to the TolC membrane protein and lipopolysaccharide. This dual-receptor strategy implies that if evolutionary dynamics are not considered, treatments with bacteriophages or bacterial toxins may lead to resistance [58]. Indeed, experiments have shown that *E. coli* can rapidly evolve TolC mutations that confer resistance to both colicin and TLS bacteriophages, highlighting the need to anticipate resistance when designing bacteriophage-based therapies.

Since this is a descriptive investigation, a deeper mechanism or functional understanding of phage biology was outside the purview. Instead, understanding the genetic links between candidate bacteriophages was critical to this work, going beyond simple phenotypic characterization. Comparative genomics, phylogenetic analyses, and synteny mapping provide insights into their evolutionary stability, functional modules, and potential host-range determinants [59]. Moreover, because bacterial resistance to bacteriophages can emerge through coevolutionary mechanisms, it is important to anticipate such dynamics when evaluating long-term efficacy [60]. The three bacteriophages isolated in this study showed characteristics that make them suitable for use in cocktails on chicken farms and in retail chains as they are small size, exclusively lytic, carriers of a depolymerase gene and they have a robust genomic safety profile with no antibiotic resistance genes or virulent genetic elements.

## 5. Conclusions

In conclusion, the bacteriophages characterized in this study exhibit genomic coherence, safe lytic profiles, biofilm-degrading potential, and host specificity aligned with the clonal structure of the Ecuadorian *S.* Infantis population, supporting their suitability for poultry-associated biocontrol applications. The next step involves evaluating their synergistic performance in a stabilized cocktail and testing their efficacy in vivo using a CDC-bioreactor under field-simulated conditions. Future work should also examine long-term phage–host evolutionary interactions and assess their stability and performance in real poultry-production matrices to facilitate their development into practical, field-ready interventions.

## Figures and Tables

**Table 1:** Genomic and taxonomic characteristics of isolated bacteriophage genomes.

Characterization		Bacteriophage_GS71	Bacteriophage_GS156	Bacteriophage_GS166
Size (bp)		48,587	48,552	48,069
GC content (%)		41.8	41.7	41.8
Coding sequences (CDS)		79	79	76
tRNA and pseudogenes		0	0	0
**Most similar by Blast**	Name	Accession number: MN994500.1 Bacteriophage NBSal001, complete genome	Accession number: PP503414.1 *Salmonella* bacteriophage Sephi301i, complete genome	Accession number: PP503414.1 *Salmonella* bacteriophage Sephi301i, complete genome
	Length (bp)	50,922	48,674	48,162
	Coverage (%)	83.00	99.00	83.00
	E-value	0.0	0.0	0.0
	Identity (%)	98.09	98.08	97.7
**Predicted Taxonomy**	Class	*Caudoviricetes*	*Caudoviricetes*	*Caudoviricetes*
	Family	*Drexlerviridae*	*Drexlerviridae*	*Drexlerviridae*
	Genus	*Tlsvirus*	*Tlsvirus*	*Tlsvirus*

**Table 2:** Predicted depolymerases in the bacteriophage genomes identified by DePP, including packaging modes determined by PhageTerm.

Bacteriophage	ORF	Start	End	Strand	Function	Product	Bacteriophage Packaging
GS71	44	23,637	27,497	+	tail	central tail fiber J	HeadFul (pac) type 1
GS156	49	24,951	28,727	+	tail	tail protein	HeadFul (pac) type 1
GS166	43	23,621	27,481	+	tail	tail protein	HeadFul (pac) type 1

## Data Availability

Accession number: PV090992.1 (*Salmonella* phage vB_GS71); Accession number: PV153712.1 (*Salmonella* phage vB_GS156); Accession number: PV090993.1 (*Salmonella* phage vB_GS166).

## Abbreviations

The following abbreviations are used in this manuscript:

ATCC	American Type Culture Collection
BLAST	Basic Local Alignment Search Tool
CDS	Coding sequence
CFU	Colony-forming unit
DLA	Double-layer agar
DNase	Deoxyribonuclease
EOP	Efficiency of plating
FDA	Food and Drug Administration
ICTV	International Committee on Taxonomy of Viruses
LB	Luria–Bertani
MDR	Multidrug-resistant
MOI	Multiplicity of infection
OD600	Optical density at 600 nm
PFU	Plaque-forming unit
PNK	Polynucleotide kinase
RefSeq	Reference Sequence
RNase	Ribonuclease
SDS	Sodium dodecyl sulfate
TEM	Transmission electron microscopy
TSB	Tryptic Soy Broth
iTOL	Interactive Tree Of Life
IQ-TREE	IQ-TREE software
VIRIDIC	Virus Intergenomic Distance Calculator
DePP	DePolymerase Predictor
